# Disrupting the cortical actin cytoskeleton points to two distinct mechanisms of yeast [*PSI*^+^] prion formation

**DOI:** 10.1371/journal.pgen.1006708

**Published:** 2017-04-03

**Authors:** Shaun H. Speldewinde, Victoria A. Doronina, Mick F. Tuite, Chris M. Grant

**Affiliations:** 1 University of Manchester, Faculty of Biology, Medicine and Health, The Michael Smith Building, Manchester, Unted Kindom; 2 Kent Fungal Group, School of Biosciences, University of Kent, Canterbury, Kent, United Kingdom; Washington University School of Medicine, UNITED STATES

## Abstract

Mammalian and fungal prions arise *de novo;* however, the mechanism is poorly understood in molecular terms. One strong possibility is that oxidative damage to the non-prion form of a protein may be an important trigger influencing the formation of its heritable prion conformation. We have examined the oxidative stress-induced formation of the yeast [*PSI*^+^] prion, which is the altered conformation of the Sup35 translation termination factor. We used tandem affinity purification (TAP) and mass spectrometry to identify the proteins which associate with Sup35 in a *tsa1 tsa2* antioxidant mutant to address the mechanism by which Sup35 forms the [*PSI*^+^] prion during oxidative stress conditions. This analysis identified several components of the cortical actin cytoskeleton including the Abp1 actin nucleation promoting factor, and we show that deletion of the *ABP1* gene abrogates oxidant-induced [*PSI*^+^] prion formation. The frequency of spontaneous [*PSI*^*+*^] prion formation can be increased by overexpression of Sup35 since the excess Sup35 increases the probability of forming prion seeds. In contrast to oxidant-induced [*PSI*^*+*^] prion formation, overexpression-induced [*PSI*^*+*^] prion formation was only modestly affected in an *abp1* mutant. Furthermore, treating yeast cells with latrunculin A to disrupt the formation of actin cables and patches abrogated oxidant-induced, but not overexpression-induced [*PSI*^+^] prion formation, suggesting a mechanistic difference in prion formation. [*PIN*^*+*^], the prion form of Rnq1, localizes to the IPOD (insoluble protein deposit) and is thought to influence the aggregation of other proteins. We show Sup35 becomes oxidized and aggregates during oxidative stress conditions, but does not co-localize with Rnq1 in an *abp1* mutant which may account for the reduced frequency of [*PSI*^*+*^] prion formation.

## Author summary

Prions are infectious agents which are composed of misfolded proteins and have been implicated in progressive neurodegenerative diseases such as Creutzfeldt Jakob Disease (CJD). Most prion diseases occur sporadically and are then propagated in a protein-only mechanism via induced protein misfolding. Little is currently known regarding how normally soluble proteins spontaneously form their prion forms. Previous studies have implicated oxidative damage of the non-prion form of some proteins as an important trigger for the formation of their heritable prion conformation. Using a yeast prion model we found that the cortical actin cytoskeleton is required for the transition of an oxidized protein to its heritable infectious conformation. In mutants which disrupt the cortical actin cytoskeleton, the oxidized protein aggregates, but does not localize to its normal amyloid deposition site, termed the IPOD. The IPOD serves as a site where prion proteins undergo fragmentation and seeding and we show that preventing actin-mediated localization to this site prevents both spontaneous and oxidant-induced prion formation.

## Introduction

Prions are infectious agents composed of misfolded proteins. They are associated with a group of neurodegenerative diseases in animals and humans that have common pathological hallmarks, typified by human Creutzfeldt-Jakob Disease (CJD). The presence of the misfolded prion protein (PrP^Sc^) underlies the development of prion diseases in a mechanism which involves conversion of the normal prion protein (PrP) into its infectious PrP^Sc^ conformation [1, 2]. Aggregated, protease-resistant PrP^Sc^ seeds are believed to act as templates that promote the conversion of normal PrP^C^ to the pathological PrP^Sc^ form, which is rich in β-sheets and resistant to chemical and enzymatic degradation. PrP^c^ can adopt an alternative conformational state by spontaneous misfolding event(s) that might be triggered by mutation, mistranslation, environmental stresses and/or by disruption of the chaperone network [3].

This ‘protein-only’ mechanism of infectivity also explains the unusual genetic behaviour of several prions found in the yeast *Saccharomyces cerevisiae* [4–8]. At present, several yeast proteins are known to form prions with many other proteins classified as potential prion candidates [9]. Additionally, the [Het-s] prion that controls vegetative incompatibility has been described in *Podospora anserina*, an unrelated fungal species [10]. [*PIN*^+^] and [*PSI*^+^] are the best studied yeast prions, which are formed from the Rnq1 and Sup35 proteins, respectively [4, 11, 12]. Sup35 is the yeast eERF3 which functions in translation termination and hence [*PSI*^+^] formation influences the recognition of translation stop codons. [*PSI*^+^] formation requires the presence of an another prion, termed [*PIN*^+^], which is often present as the prion form the Rnq1 protein whose native protein function is unknown [13–15]. However, a number of prions can be designated as [*PIN*^+^] that are required for the *de novo* formation of [*PSI*^+^] [16–18]. Several studies have demonstrated the infectious behavior of the fungal prion associated with a particular phenotype adding further weight to the ‘protein-only’ mechanism of prion propagation [19–21],

How prions form spontaneously without underlying infection or genetic change is poorly understood at the molecular level, yet if we are to develop effective preventative measures for human and animal amyloidoses, this mechanism must be established. Of particular importance is identifying what can trigger this event. Several different environmental stress conditions, including heat, oxidative and salt stresses, increase the frequency of yeast [*PSI*^+^] prion formation [22]. A number of mutants have been identified which increase the frequency of [*PSI*^+^] formation [22]. This includes a number of mutations in the protein homeostasis network including mutations in chaperones and the autophagy system [7, 23]. Additionally, formation of the yeast [*GAR*^+^] prion can be induced by bacterial exposure in a chemical induction mechanism and the [*GAR*^+^] prion can be lost upon desiccation [24–26]. The spontaneous formation of prions may therefore occur as a result of random protein misfolding events which are normally dealt with by the cellular protein quality control systems.

One strong possibility underlying the *de novo* formation of prions is that oxidative damage to the non-prion form of a protein may be an important trigger influencing the formation of its heritable prion conformation [27]. For example, methionine oxidation of mammalian PrP has been proposed to underlie the misfolding events which promote the conversion to PrP^Sc^ [28–30] while methionine oxidation destabilizes native PrP facilitating misfolding and transition to the PrP^Sc^ conformation [31]. Methionine oxidation is also a common factor in many protein misfolding diseases [32–34] and an age-dependent increase in methionine oxidation has been detected in various model systems [35]. Oxidative stress has been shown to increase the frequency of yeast [*PSI*^+^] prion formation [22]. The *de novo* formation of the [*PSI*^*+*^] prion is also significantly increased in yeast mutants lacking key antioxidants suggesting that endogenous reactive oxygen species (ROS) can trigger the *de novo* formation of the [*PSI*^*+*^] prion [36–38]. Preventing methionine oxidation by overexpressing methionine sulphoxide reductase abrogates the shift to the prion form indicating that the direct oxidation of Sup35 may trigger structural transitions favouring its conversion to the transmissible amyloid-like form [37, 38]. Hence, protein oxidation may be a common mechanism underlying the aggregation of some mammalian and some yeast amyloid-forming proteins.

The frequency of *de novo* appearance of the [*PSI*^*+*^] prion is increased by overexpression of Sup35 in [*PIN*^+^][*psi*^-^] strains which increases the probability of forming prion seeds [4]. This frequency can be influenced by components of the actin cytoskeleton which physically associate with Sup35 including various proteins of the cortical actin cytoskeleton (Sla1, Sla2, End3, Arp2, Arp3) that are involved in endocytosis [39]. Loss of some of these proteins decreases the aggregation of overexpressed Sup35 and *de novo* [*PSI*^+^] formation. This is particularly interesting given the increasing evidence suggesting that cytoskeletal structures provide a scaffold for the generation of protein aggregates. Insoluble aggregates of amyloid-forming proteins including prions are targeted to the IPOD as part of the cells’ protein quality control system [40, 41]. The IPOD is located at a perivacuolar site adjacent to the preautophagosomal structure (PAS) where cells initiate autophagy [42]. Prion conversion has been proposed to occur at the cell periphery in association with the actin cytoskeleton, prior to deposition at the IPOD [43]. The actin cytoskeleton has also been implicated in the asymmetric inheritance of oxidatively-damaged proteins [44]. Actin organization therefore appears to play an important role in the aggregation of damaged proteins, which can result in prion formation.

Oxidative stress provides a powerful tool to examine the *de novo* formation of prions since it does not necessitate overexpression or mutation of the normally soluble version of the prion protein. In this current study, we have used a mutant lacking the Tsa1 and Tsa2 antioxidants to isolate the proteins which aggregate with Sup35. We used a *tsa1 tsa2* antioxidant mutant to enrich for factors which associate with oxidized Sup35 and therefore might be important for the conversion of Sup35 to the [*PSI*^+^] prion. Our data suggest a key role for the cortical actin cytoskeleton since we identified a number of components of the Arp2/3 actin-nucleation complex which specifically associate with Sup35 in the antioxidant mutant. We show that loss of several of these factors abrogates the increased frequency of [*PSI*^+^] prion formation which is normally observed in response to oxidative stress conditions. However, these mutants do not affect the increased frequency of [*PSI*^+^] prion formation induced in response to Sup35 overexpression. We show that Sup35 oxidative damage and aggregation occurs in actin-nucleation complex mutants in response to oxidative stress conditions, but the aggregates do not appear to form normally at the IPOD. Our data suggest that the cortical actin cytoskeleton is important for the formation of a propagating [*PSI*^+^] conformer following oxidant-induced misfolding and aggregation of Sup35.

## Results

### Identification of Sup35-interacting proteins in a *tsa1 tsa2* mutant

To address the mechanism by which Sup35 forms the [*PSI*^+^] prion during oxidative stress conditions, we used tandem affinity purification (TAP) and mass spectrometry to identify proteins which associate with Sup35 in a *tsa1 tsa2* mutant. For this analysis, we used [*PIN*^+^][*psi*^-^] versions of wild-type and *tsa1 tsa2* mutant strains containing genomically-tagged Sup35. We have previously confirmed that TAP-tagging Sup35 does not affect reversible [*PSI*^+^] prion formation [37]. Freshly inoculated strains were grown for 20 hours (approximately ten generations) and Sup35 affinity-purified from both strains using TAP chromatography. The associated proteins were identified from three repeat experiments and were considered significant if they were identified in at least two independent experiments. This resulted in the identification of 63 and 47 proteins which co-purify with Sup35 in the wild-type and *tsa1 tsa2* mutant strains, respectively (S1 Table).

**Fig 1:**
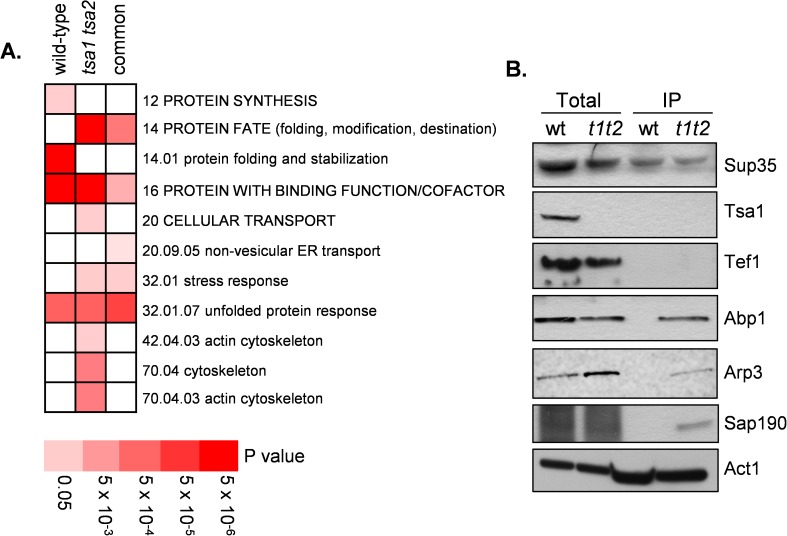
Identification of Sup35-interacting proteins in wild-type and *tsa1 tsa2* mutant strains. **A.** Functional categorisation of Sup35-associated proteins. Results are ordered on MIPS category classification numbers and overarching categories are in capitals. Where an overarching category was enriched, sub-categories within the overarching category were omitted from the graph. Confidence of each classification category is shown as Bonferroni corrected *p*-values. **B.** Sup35-TAP was immunoprecipitated from the wild-type and *tsa1 tsa2* mutant and possible interactions examined using immuno-blot analysis with the indicated antibodies. Sup35-TAP co-immunoprecipitates Abp1, Arp3 and Sap190 in a *tsa1 tsa2* mutant. Sup35-TAP co-immunoprecipitates Act1 in both wild-type and *tsa1 tsa2* mutant strains. Total denotes whole cell extracts and IP denotes immunoprecipitates.

We searched for functional categories that were enriched in the Sup35 co-purifying proteins using MIPS category classifications (Fig 1A and S2 Table). The overlap between the wild type and *tsa1 tsa2* datasets is 18 proteins and, as might be expected, this included functions related to protein fate (>3-fold enrichment; Fischer's exact test, *P*<10^−7^) such as chaperones and stress-related proteins (Sti1, Sse1, Cdc48, Ssa2, Hsc82, Ssb2, Ssa1, Hsp60) many of which have well characterized roles in prion formation and propagation in yeast [5]. Similar functional proteins were identified as part the unfolded protein response (21-fold; *P* = 1.5×10^−8^) and stress response categories (4-fold; *P* = 2×10^−5^). Over represented functions in the wild-type strain included protein synthesis (2-fold; *P* = 2×10^−5^) and protein folding and stabilization (9-fold; *P* = 2×10^−10^). The protein synthesis category included a number of ribosomal proteins and translation factors which might be expected to associate with the Sup35 translation termination factor. Interestingly, proteins associated with the actin cytoskeleton were overrepresented in the *tsa1 tsa2* mutant (8-fold; *P* = 4×10^−6^), but not in the wild-type strain and were therefore subject to further analysis.

Our data suggest an important role for the cortical actin cytoskeleton based on the cytoskeleton-related proteins which co-purify with Sup35 in the *tsa1 tsa2* mutant strain (Act1, Sac6, Crn1, Abp1, Arc40, Arp2, Arp3 and Arc35). These data strongly implicate the Arp2/3 complex, which is a seven-protein complex containing two actin-related proteins (Arp2 and Arp3) and five non-actin related proteins including Arc35 and Arc40 [45]. Abp1 is an actin-binding protein of the cortical actin cytoskeleton which is important for activation of the Arp2/3 complex [46]. Crn1 and Act1 were identified with purified Sup35 in both the wild-type and *tsa1 tsa2* mutant strains. Crn1 is an actin-binding protein which regulates the actin filament nucleation and branching activity of the Arp2/3 complex through its interaction with the Arc35 subunit [47]. Act1 is encoded by *ACT1*, a single essential gene in yeast. We validated our Sup35-interacting proteins for a number of proteins. Sup35-TAP was immunoprecipitated from the wild-type and *tsa1 tsa2* mutant and possible interactions examined using Western blot analysis (Fig 1B). This analysis confirmed that Abp1, Arp3 and Sap190 co-purify with Sup35 in the *tsa1 tsa2* mutant strain and Act1 co-purifies with Sup35 in both the wild-type and *tsa1 tsa2* mutant strains.

### Mutations disrupting the cortical actin cytoskeleton abrogate oxidative stress-induced prion formation

**Fig 2:**
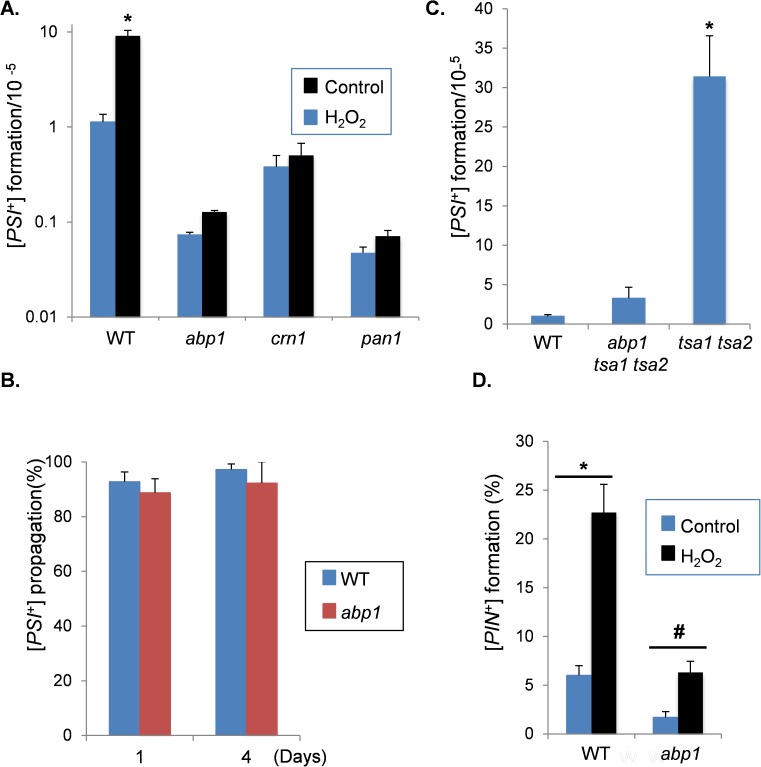
Reduced frequency of [*PSI*^+^] formation in cortical actin cytoskeleton mutants. **A.** The frequency of *de novo* [*PSI*^+^] prion formation was quantified in wild-type *abp1*, *crn1*, and *pan1* mutant strains grown in the presence or absence of 100μM hydrogen peroxide for 20 hours. [*PSI*^+^] formation was quantified using the *ade1-14* mutant allele by growth on media lacking adenine and differentiated from nuclear gene mutations by their irreversible elimination in GdnHCL. Data shown are the means of at least three independent biological repeat experiments expressed as the number of colonies per 10^5^ viable cells. Error bars denote standard deviation; * marks statistical significance at p<0.01. **B.** [*PSI*^+^] propagation was scored by culturing [*PSI*^+^]-versions of wild-type and *abp1* mutant strains for four days. [*PSI*^+^] maintenance was quantified using the *ade1-14* mutant allele as above and is expressed as the percentage of [*PSI*^+^] cells from three independent biological repeat experiments. **C.** The frequency of *de novo* [*PSI*^+^] prion formation was quantified in wild-type, *tsa1 tsa2* and *tsa1 tsa2 abp1* mutants. Error bars denote standard deviation; * marks statistical significance at p<0.01. **D.** *De novo* [*PIN*^+^] prion formation was quantified in the wild-type and *abp1* mutant strain grown in the presence or absence of 100μM hydrogen peroxide for 20 hours. Data shown are the means of three independent biological repeats expressed as the percentage of viable cells forming [*PIN*^+^] colonies. Error bars denote standard deviation (*p<0.01; # p<0.05).

In order to test whether the Sup35-associated proteins affect oxidative stress-induced prion formation, mutant strains were constructed in a [*PIN*^+^][*psi*^-^] yeast strain (74D-694) which is commonly used to study yeast prion biology. We were unable to make *arp2*, *arp3*, *arc35* or *arc40* deletion mutants in this strain background in agreement with previous observations suggesting that components of the Arp2/Arp3 complex are essential for normal growth and viability [39, 48, 49]. We therefore focused on Abp1 and Crn1, which were identified in our Sup35 immunopurification experiments. The induction of [*PSI*^+^] prion formation was quantified by analysing the formation of Ade+ colonies which arise due to nonsense suppression of the *ade1-14* mutant allele. [*PSI*^+^]-mediated suppression can be differentiated from nuclear-encoded nonsense suppressor mutations by their elimination in guanidine hydrochloride (GdnHCl). The control [*PIN*^+^][*psi*^-^] strain was grown in the presence of 100 μM hydrogen peroxide for 20 hours prior to scoring [*PSI*^+^] prion formation. This oxidative stress treatment increased the frequency of [*PSI*^+^] prion formation by approximately ten-fold (Fig 2A), similar to our previous observations [38]. The basal frequency of spontaneous [*PSI*^+^] prion formation was reduced by approximately 150-fold in the *abp1* mutant and 30-fold in the *crn1* mutant. Furthermore, loss of *ABP1* or *CRN1* abrogated the peroxide-induced increase in [*PSI*^+^] prion formation (Fig 2A). The nucleation of actin patches by the Arp2/3 complex is enhanced by the activity of nucleation promoting factors such as Abp1 [45, 50–52]. We therefore tested whether loss of another nucleation promoting factor, Pan1, similarly reduced the frequency of [*PSI*^+^] prion formation. The frequency of spontaneous [*PSI*^+^] prion formation was significantly reduced in the *pan1* mutant and no induction was observed in response to hydrogen peroxide stress (Fig 2A). We ruled out any effects on [*PSI*^+^] propagation by following [*PSI*^+^]-maintenance in [*PSI*^+^] versions of wild-type and *abp1* mutant strains. After four days of culture, the formation of [*psi*^-^] cells was comparable in the wild-type and *abp1* mutant strains (Fig 2B).

Since our Sup35-interacting proteins were identified in a *tsa1 tsa2* mutant, we examined whether deletion of *ABP1* affects the high frequency of spontaneous [*PSI*^+^] formation normally observed in a *tsa1 tsa2* mutant [36]. The frequency of [*PSI*^+^] formation was increased approximately 30-fold in a *tsa1 tsa2* mutant compared with the wild-type strain (Fig 2C). [*PSI*^+^] formation was significantly decreased in a *tsa1 tsa2 abp1* mutant suggesting that Abp1 is required for the elevated frequency of [*PSI*^+^] formation in a *tsa1 tsa2* mutant.

Since mutations which disrupt the cortical actin cytoskeleton reduce oxidative stress induced [*PSI*^+^] formation, we next examined whether the formation of another prion unrelated in sequence to the Sup35/[*PSI*^+^] prion, is similarly affected. Rnq1 can switch to the [*PIN*^*+*^] prion, which is formed at relatively high frequencies compared with the *de novo* formation of [*PSI*^*+*^] [53, 54]. The *de novo* formation of [*PIN*^+^] prions, which is also dependent on oxidative status of the cells [37], was detected in approximately 6% of control [*pin*^-^] cells, compared with 1.7% of *abp1* mutant cells (Fig 2D). Hydrogen peroxide treatment increased the frequency of [*PIN*^+^] prion formation in both strains, but the frequency of [*PIN*^+^] formation was significantly lower in the *abp1* mutant compared with the wild-type strain. Loss of *ABP1* therefore appears to decrease the spontaneous formation of both the [*PSI*^*+*^] and [*PIN*^*+*^] prions, but has a greater effect on the oxidative stress induced formation of [*PSI*^*+*^] compared with [*PIN*^*+*^].

### Loss of *ABP1*, *CRN1* or *PAN1* does not significantly alter the frequency of overexpression-induced [*PSI*^+^] prion formation

**Fig 3:**
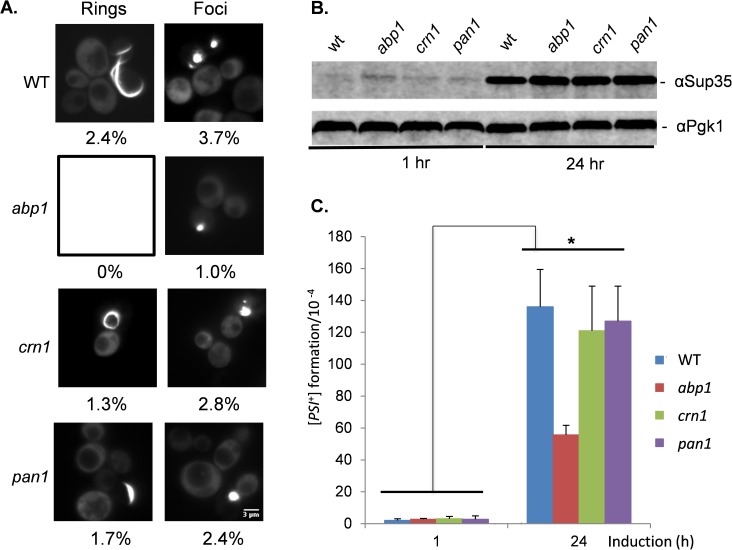
Induction of [*PSI*^+^] prion formation by overexpression of Sup35. **A.** Fluorescence micrographs are shown for [*PIN*^+^][*psi*^-^] versions of wild-type, *abp1*, *crn1* and *pan1* mutant strains containing the Sup35NM-GFP plasmid induced with copper for 24 hours. Representative Images are shown for rod/ribbon-like and foci aggregate formation. The numbers indicate the percentage of cells containing each type of aggregate from an average of 300 cells counted. The scale bar is 3μm. **B.** Western blot analysis of the wild-type and *abp1* mutant strain following induction of Sup35NM-GFP. Blots were probed with αSup35 or α-Pgk1 as a loading control **(C)** [*PSI*^+^] prion formation was quantified in the wild-type and *abp1* mutant strain containing the *Sup35NM-GFP* plasmid following 24 h of copper induction. Data shown are the means of three independent biological repeat experiments expressed as the number of colonies per 10^4^ viable cells. Error bars denote standard deviation; * marks statistical significance at p<0.01.

[*PSI*^+^] prion formation can be induced by the overexpression of Sup35 in [*PIN*^+^] [*psi*^-^] strains due to the increased possibility for prion seed formation [4]. We therefore tested whether loss of *ABP1*, *CRN1* or *PAN1* similarly affected the frequency of overexpression-induced [*PSI*^+^] prion formation. *SUP35NM-GFP* was induced for 24 hours and visible fluorescent aggregates were observed in 6.1% of wild-type cells examined (Fig 3A). This included large fluorescent foci which arise due to decorating existing aggregates (3.7%), as well as rod- and ribbon-like aggregates (2.4%) characteristic of the *de novo* formation of [*PSI*^*+*^] [39, 43, 55]. Loss of *CRN1* or *PAN1* resulted in modest decreases in Sup35 aggregation including the formation of fewer visible puncta and rod and ribbon-like aggregates (Fig 3A). A stronger effect was seen with the *abp1* mutant, with no rod or ribbon-like aggregates detected, although 1% of *abp1* mutant cells still contained visible SUP35NM-GFP puncta (Fig 3A). As a control, Western blot analysis was used to confirm that similar levels of Sup35NM-GFP were induced in the wild-type, *abp1*, *crn1* and *pan1* mutant strains (Fig 3B).

Rhodamine-phalloidin staining was used to visualize the cortical actin cytoskeleton in the wild-type and mutant strains. Multiple bright rhodamine-phalloidin–stained puncta were detected in the wild-type strain (S1 Fig), typical of the cortical actin patches normally observed in wild-type yeast cells [56, 57]. In comparison, fewer, fainter cortical actin patches were detected in the *abp1* and *crn1* mutant strains, and fewer brighter patches were detected in *pan1* mutant cells. In some cells, the formation of Sup35NM-GFP aggregates was coincident with actin patches but this was difficult to differentiate since rhodamine-phalloidin–stained puncta covered a large proportion of the cellular cortex (S1 Fig). This is similar to previous studies which have shown that few Sup35-GFP puncta [39] or no Sup35-GFP puncta [55] co-localize with actin patches. Similarly some examples of co-localized Sup35NM-GFP/rhodamine-phalloidin–stained puncta were observed in the *abp1*, *crn1* and *pan1* mutants. Although it is difficult to determine whether Sup35 aggregates are associated with the cortical actin cytoskeleton, our mutants which disrupt cortical actin patch formation do not appear to significantly affect overexpression-induced Sup35 aggregate formation.

The presence of fluorescent SUP35-GFP aggregates is not necessarily indicative of [*PSI*^+^] prion formation since some cells with fluorescent dots will die, and some contain non-productive or non-amyloid aggregates [58]. We therefore quantified [*PSI*^+^] prion formation using the *ade1-14* mutant allele as described above. [*PSI*^+^] formation was strongly induced by approximately 65-fold in the wild-type [*PIN*^+^][*psi*^-^] strain in response to Sup35 overexpression (Fig 3C). A similar induction of [*PSI*^+^] prion formation was observed in the *crn1* and *pan1* mutants. This induction was reduced in the *abp1* mutant compared with the wild-type strain, although a 21-fold increase in the frequency of [*PSI*^+^] prion formation was still observed in response to Sup35 overexpression (Fig 3C). Taken together, these data indicate that in contrast to oxidative stress-induced prion formation, mutations which disrupt cortical actin patch formation only modestly affect the induction of [*PSI*^+^] prion formation in response to Sup35 overexpression.

### Latrunculin A treatment disrupts oxidative stress-induced [*PSI*^+^] prion formation

**Fig 4:**
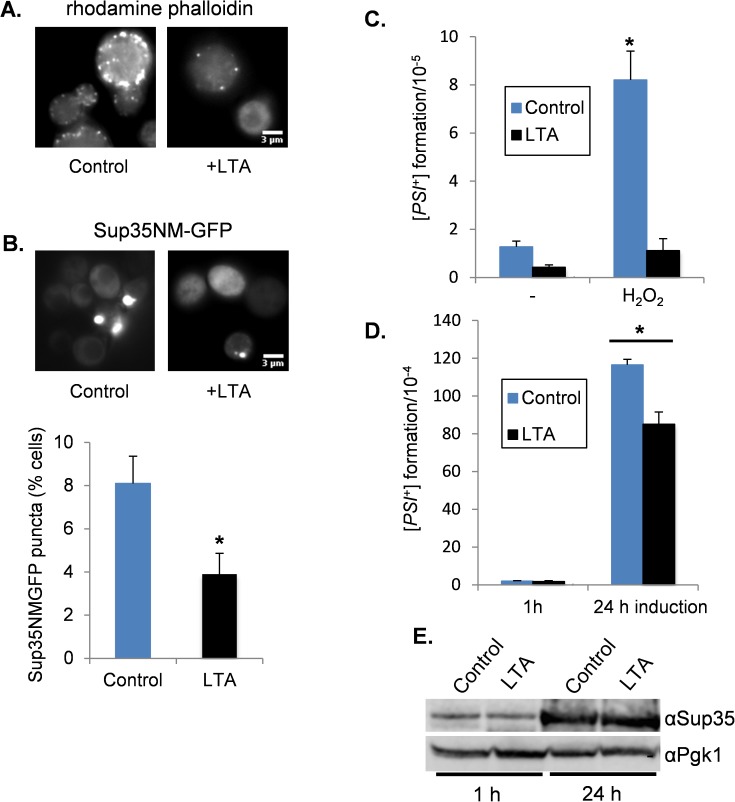
Latrunculin A treatment reduces oxidant-induced [*PSI*^+^] formation. **A.** Representative fluorescence micrographs are shown for a [*PIN*^+^][*psi*^-^] strain treated with 10 μM LTA for 20 hours. Cells were fixed, and stained with rhodamine phalloidin to visualize the cortical actin cytoskeleton. **B.** Fluorescence micrographs are shown for a [*PIN*^+^][*psi*^-^] strain treated with 100 μM hydrogen peroxide for 20 hours in the presence or absence of 10 μM LTA. The aggregate frequency is shown as the percentage of cells containing visible Sup35 foci from approximately 1000 cells counted. Error bars denote standard deviation; * marks statistical significance at p<0.01. **C.** [*PSI*^+^] prion formation was quantified in a [*PIN*^+^][*psi*^-^] strain treated with 100μM hydrogen peroxide for 20 hours in the presence or absence of 10 μM LTA. Data shown are the means of three independent biological repeats expressed as the number of [*PSI*^+^] colonies per 10^5^ viable cells. Error bars denote standard deviation; * marks statistical significance at p<0.01. **(D)** [*PSI*^+^] prion formation was quantified in a [*PIN*^+^][*psi*^-^] strain containing the *Sup35NM-GFP* plasmid following 1 and 24 h of copper induction in the presence or absence of 10 μM LTA. Data shown are the means of three independent biological repeats expressed as the number of [*PSI*^+^] colonies per 10^4^ viable cells. Error bars denote standard deviation; * marks statistical significance at p<0.01. **E.** Western blot analysis of cells grown under the same conditions as for panel D. Blots were probed with α-Sup35 and α-Pgk1 as a loading control.

Treatment of yeast cells with latrunculin A (LTA) disrupts the formation of actin cables and patches [59]. This has been used to show that the actin cytoskeleton plays a role in [*PSI*^*+*^] propagation since disrupting the actin cytoskeleton by treatment with LTA causes the loss of [*PSI*^*+*^] from yeast cells [60]. This effect was observed at relatively high concentrations of LTA (40–200 μM) and so we wanted to test whether a lower concentration of LTA, which does not significantly affect [*PSI*^*+*^] propagation, might disrupt oxidative-stress induced prion formation. We first tested the effect of growing a [*PSI*^*+*^] strain in the presence of 10 μM LTA for 20 hours. This concentration of LTA resulted in modest curing (9.3 ± 0.3%) of [*PSI*^*+*^] and so we reasoned that we could use this concentration of LTA to test whether it affects the induction of [*PSI*^*+*^]. Rhodamine-phalloidin staining was used to visualize the cortical actin cytoskeleton and to confirm that the 10 μM LTA treatment disrupted the formation of actin patches (Fig 4A).

The wild-type [*PIN*^+^][*psi*^-^]-strain was grown in the presence of 10 μM LTA and 100 μM hydrogen peroxide for 20 hours to induce prion formation. We first examined Sup35 puncta formation by expressing *SUP35NM-GFP* for the final two hours of the oxidant treatment. Approximately 8% of wild-type cells contained visible Sup35 aggregates following exposure to hydrogen peroxide. This frequency was somewhat reduced in cells treated with 10 μM LTA, where 3.9% of cells examined contained visible Sup35 aggregates (Fig 4B). [*PSI*^+^] prion formation was quantified under the same conditions, using the *ade1-14-*based assay. LTA treatment decreased the basal frequency of [*PSI*^+^] prion formation by 3-fold and also abrogated the oxidant-induced increase in the frequency of [*PSI*^+^] prion formation (Fig 4C). For comparison, we examined whether a 10 μM LTA treatment affected overexpression-induced [*PSI*^+^] prion formation. [*PSI*^+^] formation was strongly induced in response to Sup35 overexpression and only a modest decrease in induction frequency was observed in the presence of LTA (Fig 4D). Western blot analysis was used to confirm that LTA does not affect Sup35 overexpression (Fig 4E). Thus, LTA strongly disrupts oxidant-induced [*PSI*^+^] prion formation, but only modestly affects overexpression-induced [*PSI*^+^] prion formation.

### Analysis of Sup35 oxidation and protein aggregation in *abp1* mutant cells

**Fig 5:**
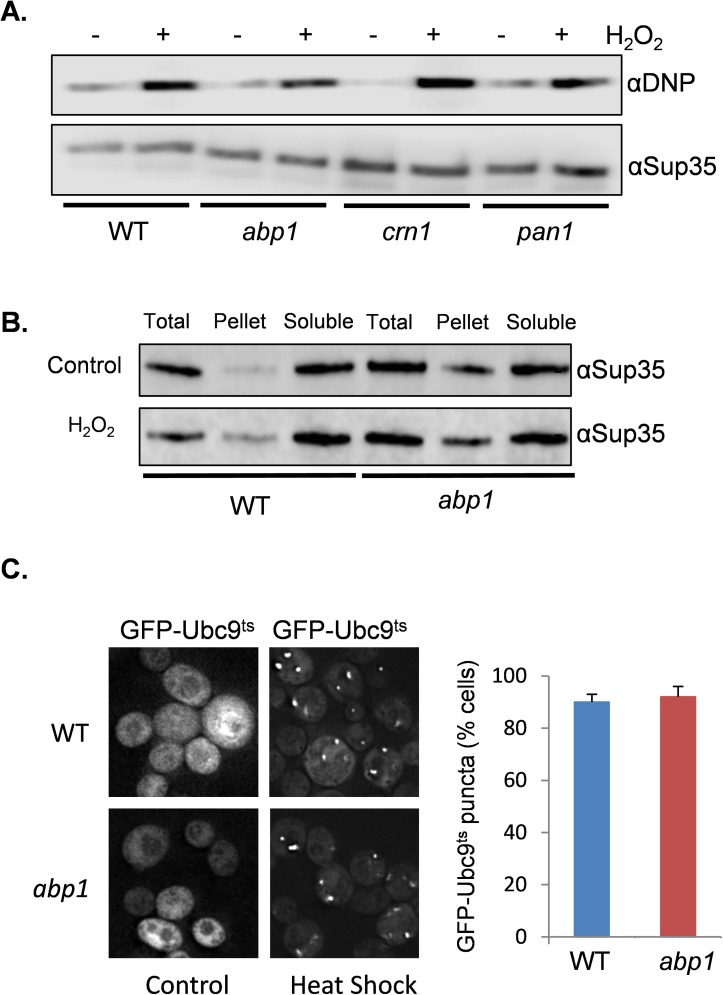
Analysis of protein oxidation and aggregation in an *abp1* mutant. **A.** Mutants disrupting the cortical actin cytoskeleton dot not affect Sup35 protein oxidative damage. The wild-type, *abp1*, *crn1* and *pan1* mutant strains were treated with 100μM hydrogen peroxide for 20 hours and protein carbonylation used as a measure of protein oxidative damage. Protein extracts were treated with the carbonyl-specific probe, DNPH, and analyzed by Western blot analysis using an antibody against DNPH. **B.** Subcellular distribution of Sup35 in wild-type and *abp1* mutant cells grown in the presence or absence of 100μM hydrogen peroxide for 20 hours. Total denotes total crude extract; Soluble, soluble fraction; Pellet, SDS-resistant high molecular weight fraction. **C.** Representative fluorescent micrographs are shown for wild-type and *abp1* mutant cells expressing GFP–Ubc9^ts^. Strains were grown in SRaf media before switching to SGal media to induce GFP–Ubc9^ts^ expression for 3 hours. This was followed by a 37°C heat shock for 30 minutes to trigger the misfolding and aggregation of Ubc9. Quantification of the frequency of GFP–Ubc9 foci is expressed as the percentage of cells containing fluorescence foci out of approximately 300 cells counted. Error bars denote standard deviation.

Disrupting the actin cytoskeleton may potentially decrease oxidative stress-induced [*PSI*^*+*^] prion formation in a number of different ways including preventing Sup35 oxidative damage, altering the formation of Sup35 protein aggregates or by disrupting the formation of heritable [*PSI*^*+*^] propagons. We have previously shown that Sup35 oxidative protein damage is an important trigger for the formation of the heritable [*PSI*^*+*^] prion in yeast [37, 38]. We therefore examined the extent of Sup35 oxidative damage in response to oxidative stress conditions, to determine whether disrupting the cortical actin cytoskeleton influences protein oxidative damage. Protein carbonylation is a commonly used measure of protein oxidative damage [61]. Carbonyl groups on proteins can be detected by Western blot analysis using an antibody against the carbonyl-specific probe DNPH. Using this assay we found that oxidative stress increased Sup35-carbonylation in response to oxidative stress as might be expected (Fig 5A). A similar increase in carbonylation was detected in the wild-type, *abp1*, *crn1* and *pan1* mutant strains suggesting that Sup35 protein oxidative damage is not altered in the mutant strains.

To address whether disrupting cortical actin patch formation influences Sup35 aggregate formation, the subcellular distribution of Sup35 was examined biochemically during oxidative stress conditions. We used a protocol which separates soluble fractions from SDS-insoluble high-molecular weight forms [62]. Sup35 was predominantly detected in the soluble fraction in wild-type cells as expected. In response to oxidative stress conditions, a small fraction of Sup35 was present in an SDS-insoluble high-molecular weight form (Fig 5B). Surprisingly, a significant proportion of Sup35 was already present in this SDS-insoluble high-molecular weight form in the *abp1* mutant in the absence of stress, and there was no further increase in response to hydrogen peroxide treatment (Fig 5B). This suggests that Sup35 aggregates in an *abp1* mutant but is not converted to the heritable [*PSI*^+^] prion form.

For comparison to Sup35 aggregation, we next examined the aggregation of a non-amyloidogenic protein in the *abp1* mutant. We used a thermolabile allele of *UBC9* fused to GFP (GFP–Ubc9^ts^) which was expressed under the control of the *GAL1* galactose-regulated promoter [63]. At permissive temperatures, GFP–Ubc9^ts^ is native and diffuse, whereas, shifting cells to 37°C causes GFP–Ubc9^ts^ to misfold and to form puncta visible by fluorescence microscopy. These protein quality control structures are referred to as Q-bodies and do not contain amyloid aggregates [63]. Cells were grown in raffinose medium prior to inducing GFP–Ubc9^ts^ expression for three hours following galactose addition. We found that approximately 90% of wild-type cells formed Q-bodies following a temperature shift to 37°C for 30 minutes (Fig 5C). A similar number of cells containing Q-bodies were also detected in the *abp1* mutant suggesting that loss of *ABP1* does not affect non-amyloidogenic protein aggregation.

### Loss of *ABP1* reduces the co-localization of Sup35 with Rnq1 puncta

**Fig 6:**
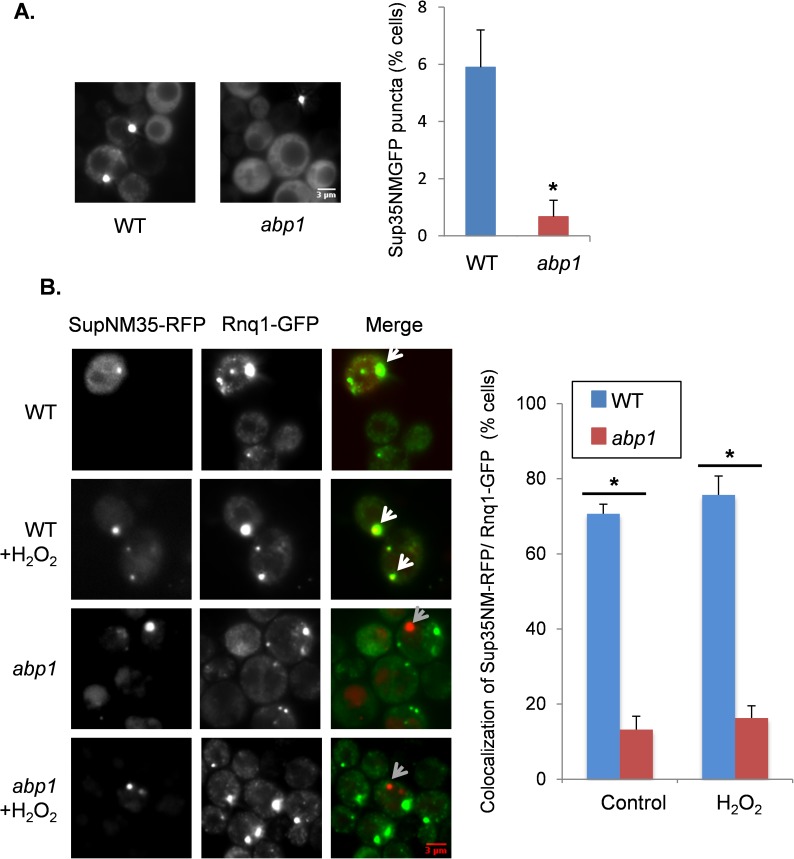
Loss of *ABP1* reduces the co-localization of Sup35 with Rnq1 aggregates. **A.** Fluorescence micrographs are shown for the wild-type and *abp1* mutant strain treated with 100 μM hydrogen peroxide for 20 hours. Sup35-NMGFP was induced with copper for 2 hours to visualize aggregate formation and the frequency is shown as the percentage of cells containing visible Sup35 foci from approximately 1000 cells counted. Error bars denote standard deviation; * marks statistical significance at p<0.01. **B.** The wild-type and *abp1* mutant strains containing Sup35NM-RFP and Rnq1-GFP were grown for 20 hours in SRaff media in the presence or absence of 100μM hydrogen peroxide before switching to SGal media for three hours to induce the expression of Sup35NM-RFP and Rnq1-GFP. White arrows show examples where Sup35NM-RFP and Rnq1-GFP co-localize, whereas, grey arrows show examples where Sup35NM-RFP does not localize with Rnq1-GFP. The frequency of co-localization of Sup35NM-RFP and Rnq1-GFP is shown as a percentage of co-localization for at least 17 Sup35NM-RFP foci scored in triplicate experiments. * marks statistical significance at p<0.01

Given that Sup35 is oxidized and aggregates in an *abp1* mutant strain we assessed the intracellular localization of Sup35 in an *abp1* mutant. We first quantified Sup35 puncta formation using the *SUP35NM-GFP* fusion to visualize aggregate formation. The wild-type and *abp1* mutant strains were treated with 100μM hydrogen peroxide for 20 hours and *SUP35NM-GFP* expression induced for the final two hours by copper addition. Following hydrogen peroxide treatment, approximately 6% of wild-type cells contained visible Sup35 aggregates (Fig 6A). This was reduced in *abp1* mutant cells where 0.5% of cells examined contained visible Sup35 aggregates.

The amyloidogenic [*PIN*^*+*^] prion form of Rnq1 localizes to the IPOD and is thought to influence the aggregation of other proteins [41, 64]. This is further supported by the observation that during overexpression induced [*PSI*^*+*^] prion formation, approximately 60% of Sup35-RFP puncta co-localize with Rnq1-GFP puncta [58]. Newly formed Sup35-RFP puncta were found to perfectly co-localize with Rnq1-GFP puncta, whereas, mature Rnq1-GFP and Sup35-RFP puncta were found that did not co-localize. We therefore visualized the relationship of Sup35 and Rnq1 during oxidant-induced [*PSI*^+^] prion formation. [*PIN*^+^][*psi*^-^]-versions of the wild-type and *abp1* mutant strains were grown in the presence of 100 μM hydrogen peroxide for 20 hours to induce prion formation. Sup35NM-RFP and Rnq1-GFP were expressed under the control of the *GAL1* promoter and were induced for three hours to visualize Sup35 and Rnq1 aggregate formation. Similar to overexpression-induced [*PSI*^*+*^] prion formation, 76% of Sup35 puncta co-localized with Rnq1 puncta following oxidant treatment of the wild-type strain (Fig 6B). In contrast, 17% of Sup35 puncta co-localized with Rnq1 puncta following oxidative stress conditions in the *abp1* mutant strain. We also searched for rare Sup35 puncta formation in the absence of stress conditions and found a similar result where 71% of Sup35 puncta colocalized with Rnq1 puncta in a wild-type strain, compared with just 13% co-localization the *abp1* mutant strain.

## Discussion

Our data suggest an important role for the Arp2/3 complex in prion formation since deletion of the *ABP1*, *CRN1* or *PAN1* genes abrogate oxidant-induced [*PSI*^+^] prion formation (Fig 2A and 2B). The Arp2/3 complex contains two actin-elated proteins (Arp2 and Arp3) and five non-actin related proteins [45]. It is required for the motility and integrity of actin cortical patches, and for actin-dependent processes such as endocytosis and organelle inheritance. For example, conditional Arp2/3 mutants are deficient in actin patch formation suggesting that Arp2/3 is required for the assembly and organization of cortical actin filaments [65]. Nucleation-promoting factors such as Abp1 and Pan1 associate with the Arp2/3 complex and stimulate actin nucleation [50, 52]. Crn1 regulates actin filament nucleation and the branching activity of the Arp2/3 complex through its interaction with the Arc35 subunit [47]. We found that disrupting the actin cytoskeleton by deletion of *ABP1*, or treating cells with LTA, decreased the frequency of [*PSI*^+^] prion formation during oxidative stress conditions (Figs 2A, 2B and 4C). This suggests that the cortical actin cytoskeleton is required for the conversion of oxidatively damaged Sup35 into its heritable prion form.

Previous studies have implicated the Arp2/3 complex in prion formation and shown that Sup35 physically interacts with various proteins of the cortical actin cytoskeleton [39]. This includes Arp2 and Arp3 which were shown to interact with the N-terminal prion-forming domain of Sup35 during normal non-stress conditions using a two-hybrid assay. In contrast, we found that Arp2 and Arp3 are enriched in the Sup35-interacting proteins identified under oxidative stress conditions (Fig 1). This difference might arise since we have used native Sup35 expressed under the control of its own promoter, rather than a fragment of Sup35 fused to the DNA domain of Gal4 in a two-hybrid assay. Additionally, Gal4-activation domain fusion proteins are unlikely to assemble into normal actin complex structures in the nuclear two-hybrid assay [39]. An interaction between actin and overexpressed Sup35 was also demonstrated using immunoprecipitation experiments [39] similar to our finding that Sup35 interacts with actin in wild-type and *tsa1 tsa2* mutant cells (Fig 1). We also found that Crn1 interacts with Sup35 in both the wild-type and *tsa1 tsa2* mutant strains. Arp2/3 complex-related proteins therefore appear to be common Sup35-interacting proteins, although certain components show an increased interaction under oxidative stress conditions.

Disrupting the cortical actin cytoskeleton, either genetically by deletion of *ABP1*, *CRN1* or *PAN1*, or chemically, by treating cells with LTA, prevented oxidative stress-induced [*PSI*^+^] prion formation. In contrast, similar disruption of the cortical actin cytoskeleton did not significantly alter Sup35-overexpression-induced [*PSI*^+^] prion formation (Figs 3C and 4D). This suggests that the mechanism underlying the conversion of the soluble protein to its amyloid form is different for overexpression-induced versus oxidative stress-induced prion formation. However, there are mechanistic similarities for these two induction pathways since cortical actin cytoskeleton mutants do influence overexpression-induced [*PSI*^+^] prion formation. For example, an *abp1* mutant abrogated Sup35 ring formation, although the frequency of [*PSI*^+^] prion formation was still strongly induced in this mutant. Other studies have linked cortical actin patch formation with overexpression-induced [*PSI*^+^] prion formation. For example, loss of *LAS17* or *SAC6* abrogates overexpression-induced [*PSI*^+^] prion formation [66, 67]. Las17 is a nucleation-promoting factor similar to Pan1 and Abp1, although Las17 is a stronger nucleation-promoting factor compared with Pan1 and Abp1 [68]. Sac6, an actin-bundling protein, is the major F-actin crosslinking protein in budding yeast [69]. Additionally, loss of other genes affecting actin patch formation including *SLA1*, *SLA2* and *END3* decreases the frequency of Sup35 overexpression-induced [*PSI*^+^] formation [39, 43]. Taken together, these data strongly implicate actin cytoskeletal networks in *de novo* [*PSI*^+^] prion formation, although there appear to be mechanistic differences between overexpression and oxidative stress-induced prion formation.

Much previous research has made use of the Sup35NM-GFP fusion that we used to visualize [*PSI*^+^] prion formation. As with wild-type Sup35, the Sup35NM-GFP fusion protein retains the unstructured PrD that has a high propensity to misfold [39, 55, 58, 70]. Overexpression of Sup35NM-GFP is frequently used to visualize [*PSI*^+^] formation since the spontaneous formation of prions is very low making it difficult to observe during normal growth conditions. Oxidative stress conditions increase the frequency of [*PSI*^+^] formation making it possible to visualize [*PSI*^+^] formation without Sup35 overexpression. We observed cells containing puncta, which included examples of cells containing few (sometimes one) large dots and cells containing multiple smaller dots (Fig 6). The frequency of puncta formation was reduced in the *abp1* mutant compared with a wild-type strain, although it was still significantly higher than the frequency of [*PSI*^*+*^] prion formation observed in the *abp1* mutant in response to the same oxidative stress conditions. This is not surprising because Sup35-GFP puncta are not necessarily indicative of amyloidogenic-aggregation since GFP-puncta may arise due to amorphous aggregation or the formation of other granules such as stress granules. In fact, cell fractionation experiments revealed that Sup35 was more prevalent in an SDS-insoluble high-molecular weight form in an *abp1* mutant during both non-stress and oxidative stress conditions compared with a wild-type strain (Fig 5B). Together with our observation that Sup35 protein oxidation is similar in wild-type and *abp1* mutant strains (Fig 5A) it does not seem likely that alterations in protein oxidation and aggregation account for the reduced frequency of [*PSI*^+^] prion formation in cortical actin cytoskeleton mutants. The Arp2/3 complex may therefore be required to provide the driving force for aggregate movement via growing actin filament formation. In the absence of the Arp2/3 complex, Sup35 aggregates are formed but are not transported to protein quality control compartments where prion formation occurs.

Accumulating evidence suggests that eukaryotic cells defend themselves against protein aggregation by sequestering misfolded proteins into defined quality control compartments. Studies in yeast cells have revealed intricate protein quality control systems where insoluble proteins are partitioned into defined sites in the cell. Amyloid and amorphous aggregates are believed to be processed via distinct cytosolic protein inclusion bodies [40, 41]. Upon proteasome inhibition, JUNQ serves as a sequestration site for ubiquitinated proteins, whereas, the IPOD sequesters terminally misfolded and amyloidogenic proteins. When proteasomes are active, misfolded proteins aggregate into Q-bodies [63, 71, 72]. Overexpressed Sup35NM-GFP is initially soluble, but the PrD has a high propensity to misfold and the misfolded protein is thought to be targeted to the IPOD via Myo2-based actin cable transport [64, 73]. Other prion proteins including Ure2 and Rnq1 localize to the IPOD which facilitates nucleation and [*PSI*^+^] induction [41, 64]. Accordingly, following Sup35NM-GFP overexpression, most newly induced Sup35 dots overlap with Rnq1 dots [58]. Similarly, we found that most oxidant-induced Sup35 dots co-localized with Rnq1 dots in a wild-type strain (Fig 6). In contrast, relatively few oxidant-induced Sup35 dots were found to co-localize with Rnq1 dots in an *abp1* mutant. This may explain the reduced frequency of [*PSI*^+^] prion formation since Sup35 forms aggregates in response to oxidative stress conditions, but these aggregates do no efficiently localize to the IPOD in an *abp1* mutant and hence do not form heritable propagons.

The IPOD is formed on a perivacuolar site adjacent to the pre-autophagosome (PAS) where cells initiate autophagy [40, 64]. One possibility is that the PAS may serve to recruit aggregated prion proteins prior to autophagic turnover. Previous studies may be complicated by overexpressing Sup35NM-GFP to follow [*PSI*^*+*^] formation which might overwhelm or impair autophagic flux, and it has been suggested that the IPOD provides a storage site for excess aggregates [73]. It is known that autophagy protects against *de novo* formation of [*PSI*^*+*^] and [*PIN*^+^], and conversely, increasing autophagic flux by treating cells with the polyamine spermidine suppresses prion formation in mutants which normally show a high frequency of *de novo* prion formation [23, 74]. Growth under anaerobic conditions in the absence of molecular oxygen prevents Sup35 protein damage and suppressed the high frequency of [*PSI*^+^] formation in an autophagy mutant further reinforcing the idea that oxidatively damaged Sup35 is cleared by autophagy to protect against the structural transitions favouring its conversion to the propagatable [*PSI*^+^] form [23].

Disrupting the cortical actin cytoskeleton not only prevented oxidant-induced [*PSI*^+^] formation, but also reduced the spontaneous frequency of *de novo* [*PSI*^+^] formation (Fig 2A). Similarly, the co-localization of Sup35 and Rnq1 was also disrupted in an *abp1* mutant during non-stress conditions (Fig 6B). Mammalian and fungal prions arise *de novo* and for example sporadic forms of CJD account for approximately 80% of all recognised prion disease. However, the mechanism is poorly understood in molecular terms. It is therefore tempting to speculate that oxidant-induced prion formation provides a model for these spontaneous events. Elucidating the underlying mechanism of *de novo* prion formation following protein oxidation and how it depends on the balance between clearance of misfolded proteins mediated by autophagy and the formation of transmissible propagons, will enable a more mechanistic understanding of *de novo* prion formation.

## Materials and methods

### Yeast strains and plasmids

The wild-type yeast strain 74D-694 (*MATa ade1-14 ura3-52 leu2-3*,*112 trp1-289 his3-200)* was used for all experiments. Strains deleted for *TSA1* (*tsa1*::*LEU2*) and *TSA2* (*tsa2*::*kanMX*) and containing Sup35 tagged at its C-terminus with a tandem affinity purification (TAP) tag have been described previously [37]. Strains deleted for *ABP1*, *CRN1* and *PAN1* were constructed in 74D-694 using standard yeast methodology.

The yeast plasmid *CUP1-SUP35NM-GFP* [*URA3*] expressing the Sup35NM domain conjugated to GFP under the control of the *CUP1* promoter has been described previously [75] as has the yeast plasmid p2018 containing *GAL1-SUP35NM-RFP* [*LEU2*] [58]. Rnq1 was visualized using a yeast plasmid containing *GAL1-RNQ1-EGFP* [*URA3*] which expresses Rnq1-GFP under the control of the *GAL1* promoter [76]. The yeast plasmid expressing RFP-tagged Hsp104 (pRP1186, Hsp104-RFP) has been described previously [77]. A thermo-labile allele of *UBC9* fused to GFP (*GFP–Ubc9*^ts^) was expressed under the control of the *GAL1* galactose-regulated promoter [63]

### Growth and stress condition

Strains were grown at 30°C with shaking at 180 rpm in rich YEPD medium (2% w/v glucose, 2% w/v bactopeptone, 1% w/v yeast extract) or minimal SD (0.67% w/v yeast nitrogen base without amino acids, 2% w/v glucose) supplemented with appropriate amino acids and bases. SRaf media contained 2% w/v raffinose and SGal media contained 2% w/v galactose. Media were solidified by the addition of 2% (w/v) agar. Strains were cured by five rounds of growth on YEPD agar plates containing 4 mM guanidine hydrochloride (GdnHCl). Where indicated, strains were grown in the presence of 100 μM hydrogen peroxide for 20 hours prior to analysing [*PSI*^+^] prion formation. Cells were treated with 10 μM latrunculin to disrupt the actin cytoskeleton.

### Analyses of prion formation

The frequency of spontaneous [*PSI*^+^] prion formation was scored by growth in the absence of adenine. Diluted cell cultures were plated onto SD plates lacking adenine (SD-Ade) and incubated for 7–10 days. Colonies which grew on SD-Ade plates were counted and then picked onto new SD-Ade plates before replica-printing onto SD-Ade and SD-Ade containing 4mM GdnHCl. Colonies that grew on SD-Ade, but not on SD-Ade with GdnHCl were scored as [*PSI*^+^]. [*PSI*^+^] colonies were also scored by visual differentiation of red/white colony formation on YEPD plates and by the conversion of pink/white [*PSI*^+^] colonies to red [*psi*^-^] colonies on YEPD plates containing GdnHCl. For oxidant induced prion assays, cultures were grown in the presence of 100 μm hydrogen peroxide for 20 hours prior to scoring [*PSI*^+^] formation. For Sup35 overexpression-induced prion assays, cultures were grown in the presence of 50 μM copper sulphate for 20 hours to induce *CUP1-SUP35NM-GFP* expression prior to scoring [*PSI*^+^] formation [23]. *De novo* [*PIN*^*+*^] formation was performed as previously described [37]. Briefly, Sup35NM-GFP was overexpressed in [*pin*^–^] [*psi*^–^] strains in order to detect cells that generate [*PSI*^+^] *de novo*. Since [*PSI*^*+*^] formation is dependent on cells being [*PIN*^*+*^] [53], the rate of [*PIN*^*+*^] formation was estimated based on the number of [*PSI*^+^] cells which arise. [*PSI*^+^] and [*PIN*^*+*^] formation was calculated based on the means of at least three independent biological repeat experiments.

### Microscopy analysis

Rhodamine phalloidin staining of actin was performed as described previously [78]. Sup35 aggregate-formation was visualized using *CUP1-SUP35NM-GFP* following 50 μM copper sulphate addition to induce the *CUP1* promoter [37]. Sup35 and Rnq1 co-localization experiments were conducted using plasmids containing *GAL1-SUP35NM-RFP* and *GAL1-RNQ1-EGFP*. Strains were grown in the presence of absence of 100μM hydrogen peroxide for 20 hours in SRaf media before switching to SGal media for three hours to induce the expression of *GAL-SUP35NM-RFP* and *GAL1-RNQ1-EGFP*. Visualization of the aggregation of a non-amyloidogenic protein, Ubc9, was performed using *GAL1-GFP–Ubc9*^*ts*^. Strains were grown in SRaf media before switching to SGal media for 3 hours to induce the expression of *GAL1-GFP–Ubc9*^*ts*^. The temperature was shifted to 37°C for the final 30 minutes to trigger Ubc9 misfolding.

Cells were washed and immobilised on 10% poly-L-lysine-coated slides. All images were acquired on a Delta Vision (Applied Precision) restoration microscope using a 100x/NA 1.42 Plan Apo objective and fluorescein isothiocyanate (FITC) and Texas Red band pass filters from the Sedat filter set (Chroma). The images were collected using a Coolsnap HQ (Photometrics) camera with a Z optical spacing of 0.2μm. Raw images were then deconvolved using the Softworx software and maximum intensity projections of these deconvolved images are shown in the results.

### Sup35-TAP affinity purification and mass spectrometry

Sup35-TAP affinity purification was performed as described previously [37]. Sup35-interacting proteins were identified in the wild-type and *tsa1 tsa2* mutant by mass spectrometry in triplicate for each strain. For protein identification, protein samples were run a short distance into SDS-PAGE gels and stained using colloidal Coomassie blue (Sigma). Total proteins were excised, trypsin digested, and identified using liquid chromatography-mass spectrometry (LC-MS) performed by the Biomolecular Analysis Core Facility, Faculty of Biology, Medicine and Health, University of Manchester. Proteins were identified using the Mascot mass fingerprinting programme (www.matrixscience.com) to search the NCBInr and Swissprot databases. Final datasets for each condition were determined by selecting proteins that were identified in at least two of the three replicates.

### Protein analysis

Protein extracts were electrophoresed under reducing conditions on SDS-PAGE minigels and electroblotted onto PVDF membrane (Amersham Pharmacia Biotech). Bound antibody was visualised by chemiluminescence (ECL, Amersham Pharmacia Biotech). Primary antibodies used were Sup35 [62], Tsa1 [79], Tef1 [80], Abp1 (abcam), Arp3 (Santa Cruz Biotechnology), Sap190 (affinity-purified polyclonal antibody raised against Sap190 peptides), Act1 (ThermoFisher Scientific) and Pgk1 (ThermoFisher Scientific).

The analysis of Sup35 aggregates by subcellular fractionation was performed essentially as described previously [62]. Briefly, exponential phase cells (*A*_600_ ∼0.5) were collected by centrifugation, washed once with distilled water, and resuspended in buffer ST (10mM sodium phosphate buffer, pH7.5, 250mM NaCl, 2% w/v SDS, 1% w/v Triton X-100, 2mM PMSF). Cells were broken with glass beads using a Minibead beater (Biospec Scientific, Bartlesville) for 30 s at 4°C and centrifuged at 3000g for 3 minutes at 4°C. The supernatant (total) was centrifuged at 13000g for 45 minutes at 4°C to separate the soluble (supernatant) and insoluble (pellet) fractions. The soluble and insoluble fractions were resuspended in an equal volume of ST buffer prior to western blot analysis.

Protein carbonylation was measured by reacting carbonyl groups with 2,4-dinitrophenyl-hydrazine (DNPH) based on previously described methods [81, 82]. Briefly, exponential phase cells (*A*_600_ ∼0.5) were broken with glass beads in 10% trichloroacetic acid (TCA) using a Minibead beater. The supernatant was centrifuged at 13000g for 15 mins at 4°C and the protein pellet washed with acetone to remove residual TCA. The pellet was dried and resuspended in 70 μl of 6% (w/v) SDS. 70 μl of 10mM 2,4-dinitrophenyl hydrazine (DNPH) in 10% trifluoroacetic acid was added and incubated at room temperature for 20 minutes. 45 μl of 2 M Tris/30% glycerol was added to the suspension and mixed to neutralize the DNPH reaction. SDS-PAGE sample buffer was added prior to western blot analysis using rabbit anti::DNPH (Dako) antibodies to detect carbonylation.

### Statistical analysis

Data are presented as mean values ± standard deviation (SD). Statistical analysis for multiple groups was performed using one-way ANOVA with pair-wise comparisons of sample means via the Turkey HSD test. An unpaired two-tailed t-test was used for statistical analyses of two groups of samples. Results were considered statistically significant with a *p*-value less than 0.05.

## Supporting information

S1 Fig Visualization of overexpression-induced Sup35 aggregates and the cortical actin cytoskeleton. Fluorescence micrographs are shown for [*PIN*^+^][*psi*^-^] versions of the wild-type, *abp1*, *crn1* and *pan1* mutant strains containing the Sup35NM-GFP plasmid induced with copper for 24 hours. Rhodamine-phalloidin staining was used to visualize the cortical actin cytoskeleton.
(TIF)

S1 Table Proteins co-purifying with Sup35 in wild-type and *tsa1 tsa2* mutant strains. Sup35-TAP was immunoprecipitated from the wild-type and *tsa1 tsa2* mutant strains and the associated proteins identified from three repeat experiments using mass spectrometry. This resulted in the identification of 63 and 47 proteins which are shown according to whether they were identified in the wild-type, *tsa1 tsa2* mutant or both strains.
(DOCX)

S2 Table Functional categorisation of Sup35-associated proteins. The proteins identified within each MIPS category classification shown in Fig 1A are listed.
(XLSX)
