# Supplementary figures and images for: Disrupting the cortical actin cytoskeleton points to two distinct mechanisms of yeast [PSI+] prion formation
Source: PLoS Genet. 2017 Apr 3;13(4):e1006708. doi: 10.1371/journal.pgen.1006708 (PMC5393896; doi:10.1371/journal.pgen.1006708)

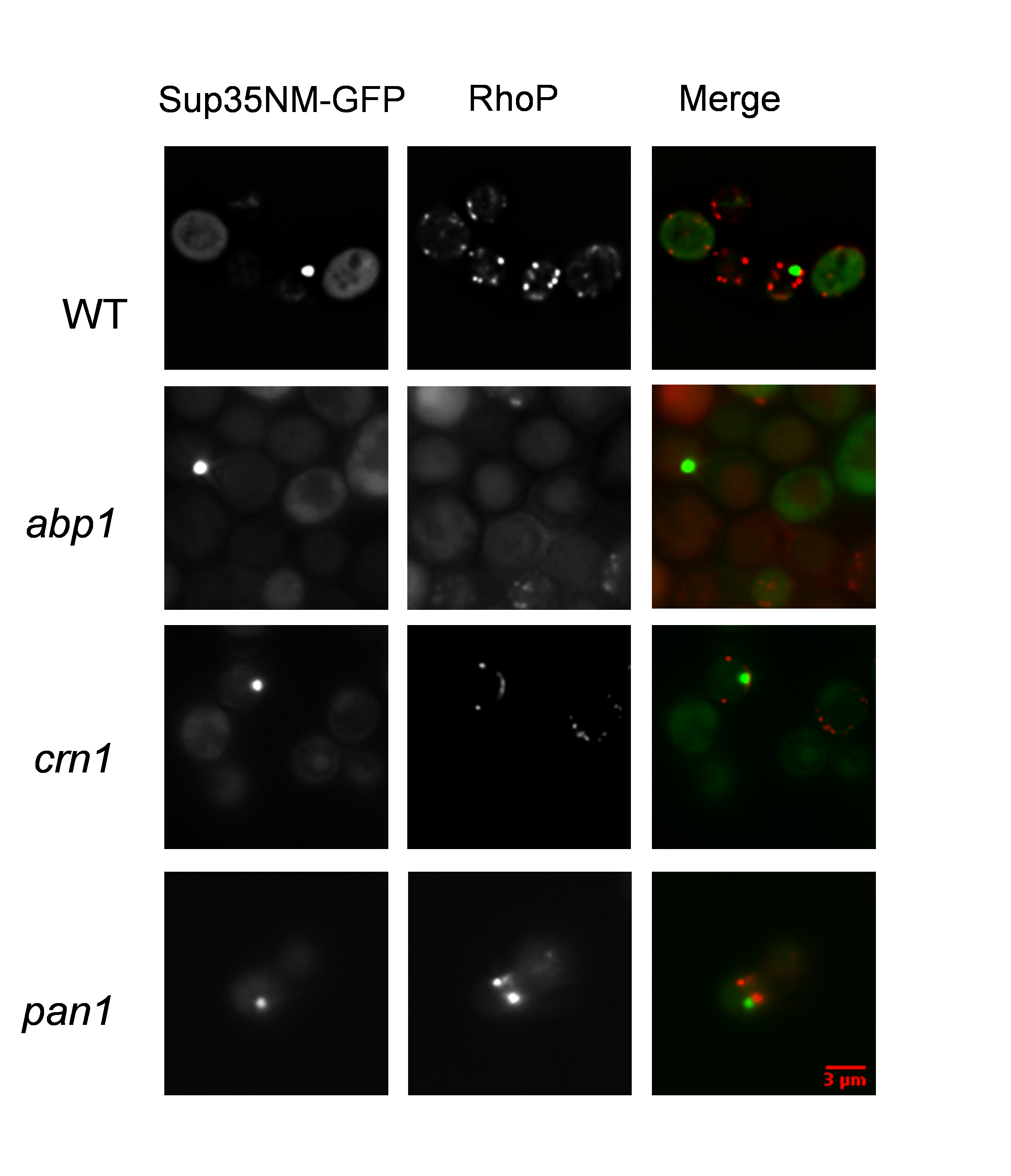

Supplement: S1 Fig — Fluorescence micrographs are shown for [PIN+][psi-] versions of the wild-type, abp1, crn1 and pan1 mutant strains containing the Sup35NM-GFP plasmid induced with copper for 24 hours. Rhodamine-phalloidin staining was used to visualize the cortical actin cytoskeleton. (TIF) [file pgen.1006708.s001.tif]
