# Supplementary material for: Disrupting the cortical actin cytoskeleton points to two distinct mechanisms of yeast [PSI+] prion formation
Source: PLoS Genet. 2017 Apr 3;13(4):e1006708. doi: 10.1371/journal.pgen.1006708 (PMC5393896; doi:10.1371/journal.pgen.1006708)
Supplement: S1 Table — Sup35-TAP was immunoprecipitated from the wild-type and tsa1 tsa2 mutant strains and the associated proteins identified from three repeat experiments using mass spectrometry. This resulted in the identification of 63 and 47 proteins which are shown according to whether they were identified in the wild-type, tsa1 tsa2 mutant or both strains. (DOCX) [file pgen.1006708.s002.docx]

**S1 Table. Proteins co-purifying with Sup35 in wild-type and *tsa1 tsa2* mutant strains**

| Strain | Protein |
| --- | --- |
| Wild-type (63) | Acc1, Act1, Adh1, Atp1, Ccd48, Cct8, Cdc19, Crn1, Ddr48, Eft1, Eno2, Faa1, Faa4, Fpr3, Gcd6, Gcn1, Gfa1, Hsc82, Hsp60, Ilv2, Kap123, Kar2, Mcm6, Mss116, Nan1, Nop56, Nop58, Pdi1, Pet9, Pfk1, Pma1, Prp43, Puf6, Rpl13B, Rpl21A, Rpl3, Rpl4A, Rpl6Bm, Rpn2, Rps20, Rps6B, Rrp5, Rvb2, Sam1, Sec23, Sec26, Ssa1, Ssa2, Ssb2, Ssc1, Sse1, Sst1, Tdh1, Tdh2, Tef2, Tub2, Ura1, Ura7, Utp4, Utp8, Vma1, Vma2, YHR020W |
| *tsa1 tsa2* (47) | Abp1, Acc1, Act1, Aif1, Arc35, Arc40, Arp2, Arp3, Asc1, Cdc48, Cop1, Crn1, Eft1, Fas1,, Fas2, Hsc82, Hsp104, Hsp60, Hsp78, Kap123, Lpd1, Noc3, Pdi1, Pim1, Pma1, Prc1, Rpb2, Rpn2, Sac6, Sap185, Sap190, Sec21, Sec26, Sec27, Spt5, Ssa1, Ssa2, Ssa4, Ssb2, Sse1, Sti1, Tef2, Ufd4, Ufo1, Ura2, Vps13, Yef3 |
| Common (18) | Acc1, Act1, Cdc48, Crn1, Eft1, Hsc82, Hsp60, Kap123, Pdi1, Pma1, Rpn2, Sec26, Ssa1, Ssa2, Ssb2, Sse1, Sti1, Tef2 |
